# Maternity and Mothering

**Published:** 1911-12-16

**Authors:** 


### MATERNITY AND MOTHERING.

**Inquiries and Correspondence are invited.**

### Nocturnal Incontinence.

Among the childish ailments and troubles that vex the minds of anxious mothers and of all those who have the care of children, there are few which are so distressing and so refractory to treatment as incontinence of urine. As every practitioner gets to know, the worry and mental anguish of mothers whose children are thus affected is out of all proportion to the magnitude of the mischief.

### Inhibition an Acquired Power.

Ability to control the passage of urine is, of course, an acquired power; in the nursling it does not exist. Moreover, the development of this control is gradual and at first somewhat irregular. It is therefore not possible to lay down any hard and fast limit or standard to which all babies should conform; but in general terms it may be said that in the majority of children full control, both waking and sleeping, is gained before the age of two and a half years—often long before—and that failure to control micturition after that age constitutes incontinence.

Incontinence of urine in children varies enormously in degree. At one end of the scale are those who merely micturate once or twice a week during sleep up to the age of three or four years; at the other those whose life is rendered miserable, even to adolescence or adult age, by unconquerable sphincter weakness, waking and sleeping. The former class is not separable by any hard and fast line from children who may be deemed normal; the latter's plight is perhaps as pitiable as it is extremely rare.

The cases of a moderate degree of incontinence are, however, not by any means rare. Usually the failure of control is nocturnal only—that is, incontinence occurs only during sleep, and then only after several hours of it. For such children the remedies which have been suggested are innumerable, and this is evidence at once of the intractability of the condition and of the inefficiency of most remedies. First of all, the assistance of a medical man is essential, though his efforts depend largely for success upon the constant and intelligent co-operation of the mother and nurse. One reason why no parent should undertake to treat a child for incontinence without a doctor, is that in some cases conditions exist which must be remedied before there is any prospect of success in curing the incontinence—the condition known as phimosis is one of these, and there are several others. Assuming that such obstacles have been removed, if they exist, what are the next principles of treatment?

First and foremost, never punish the child for "being dirty." The occurrence is one over which he (or she) has no control, and any kind of severity is only likely to accentuate the trouble by making the child nervous. Next the intake of fluids should be limited during the three hours preceding the ordinary bedtime; the patient must not be deprived of all fluid, or allowed to suffer from thirst, but curtail the fluid element of the food. An effort should be made to find out at what time the incontinence takes place; and the practice should be instituted of waking the child an hour before this time and of commanding it to micturate. It used to be supposed that sleeping on the back has something to do with enuresis, and devices were invented to prevent this. Unfortunately they are more or less cruel, and in addition there is little proof of any great utility; one of the favourite measures was to fix a cotton-reel in the small of the back by a tape passed round the waist. Some doctors believe that adenoids and incontinence are somehow associated; and cases have been reported in which removal of adenoids has been followed by cure of incontinence. Certainly a child with adenoids enjoys better health in every way after proper surgical treatment; and therefore removal of definite adenoids is always likely to be beneficial. But it seems doubtful whether there is any strictly causal connection between the two ailments, and certainly no child should be submitted to operation on the nose because it has incontinence, if the adenoid condition does not warrant interference.

### The First Drug to Try.

Last of all comes medicinal treatment. Of all the drugs with which physicians have dosed the sufferers from incontinence, there was only one with which any frequent success was obtained; that one was atropine, the alkaloid of belladonna. Fortunately children stand atropine well, for it is generally necessary to give large doses for a considerable period of time, and even then the drug often fails. Two years ago Dr. Leonard Williams described remarkable improvement in cases of incontinence following the regular administration of thyroid extract, and suggested that hypothyroidism may be the real explanation of the condition. Other physicians have failed to obtain results as brilliant, and atropine still remains the favourite drug for first trial.

In a recent paper Dr. A. C. D. Firth * records his impressions of the thyroid treatment of twenty-eight cases of inveterate nocturnal incontinence. Briefly, he noticed cure or marked improvement in sixteen patients, and total failure in twelve. Although the doses given were slightly smaller than those recommended by Dr. Williams, symptoms of hypothyroidism were caused in five cases, and hence it may be assumed that the drug was pushed to the utmost permissible limit. The twelve children who failed to improve on thyroid treatment were then given atropine, and five of them showed marked improvement. Dr. Firth's analysis of his cases shows that thyroid extract is most likely to do good when the child is backward in other ways—that is, when there is confirmatory evidence of hypothyroidism. In children otherwise normal, atropine holds out the best prospect of cure.

* *Lancet*, December 9, 1911, p. 1619.

